# Development of Marine-Derived Compounds for Cancer Therapy

**DOI:** 10.3390/md19060342

**Published:** 2021-06-15

**Authors:** Weimin Zuo, Hang Fai Kwok

**Affiliations:** 1 Cancer Centre, Faculty of Health Sciences, University of Macau, Avenida de Universidade, Taipa, Macao; yc07632@umac.mo; 2 MoE Frontiers Science Center for Precision Oncology, University of Macau, Taipa, Macao

**Keywords:** marine-derived compounds, cancer therapy, mechanism, technology

## Abstract

Cancer has always been a threat to human health with its high morbidity and mortality rates. Traditional therapy, including surgery, chemotherapy and radiotherapy, plays a key role in cancer treatment. However, it is not able to prevent tumor recurrence, drug resistance and treatment side effects, which makes it a very attractive challenge to search for new effective and specific anticancer drugs. Nature is a valuable source of multiple pharmaceuticals, and most of the anticancer drugs are natural products or derived from them. Marine-derived compounds, such as nucleotides, proteins, peptides and amides, have also shed light on cancer therapy, and they are receiving a fast-growing interest due to their bioactive properties. Their mechanisms contain anti-angiogenic, anti-proliferative and anti-metastasis activities; cell cycle arrest; and induction of apoptosis. This review provides an overview on the development of marine-derived compounds with anticancer properties, both their applications and mechanisms, and discovered technologies.

## 1. Introduction

According to *Global Cancer Statistics 2020*, cancer remains a huge burden worldwide [1]. Among all the cancer types, breast cancer is the most common and lethal cancer among females, while lung cancer is the most common and the most lethal cancer among males. In 2020, an occurrence of about 19.3 million new cancer cases and almost 10.0 million cancer deaths were estimated worldwide [1]. In America, about 1.9 million new cancer cases and approximately 0.6 million cancer deaths were estimated to occur in 2021 [2]. While in China, about 4.3 million new cancer cases and 2.9 million cancer deaths were estimated to occur in 2018 [3]. To understand the biology of cancer, Douglas Hanahan and Robert A. Weinberg have proposed 10 hallmarks of cancer, including evading growth suppressors, avoiding immune destruction, enabling replicative immortality, tumor-promoting inflammation, activating invasion and metastasis, inducing angiogenesis, genome instability and mutation, resisting cell death, deregulating cellular energetics and sustained proliferative signaling [4]. Many drugs were used to target these hallmarks, such as EGFR inhibitors, VEGF signaling inhibitors, cyclin-dependent kinase inhibitors and immune activating antibodies [5]. However, cancer still remains a heavy burden because of its recurrence, the resistance to drugs and the drugs’ side effects.

**Figure 1:**
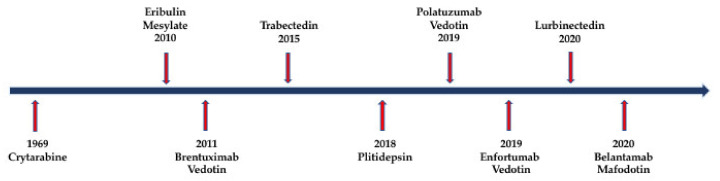
Timeline of the approval of marine-derived drugs.

**Figure 2:**
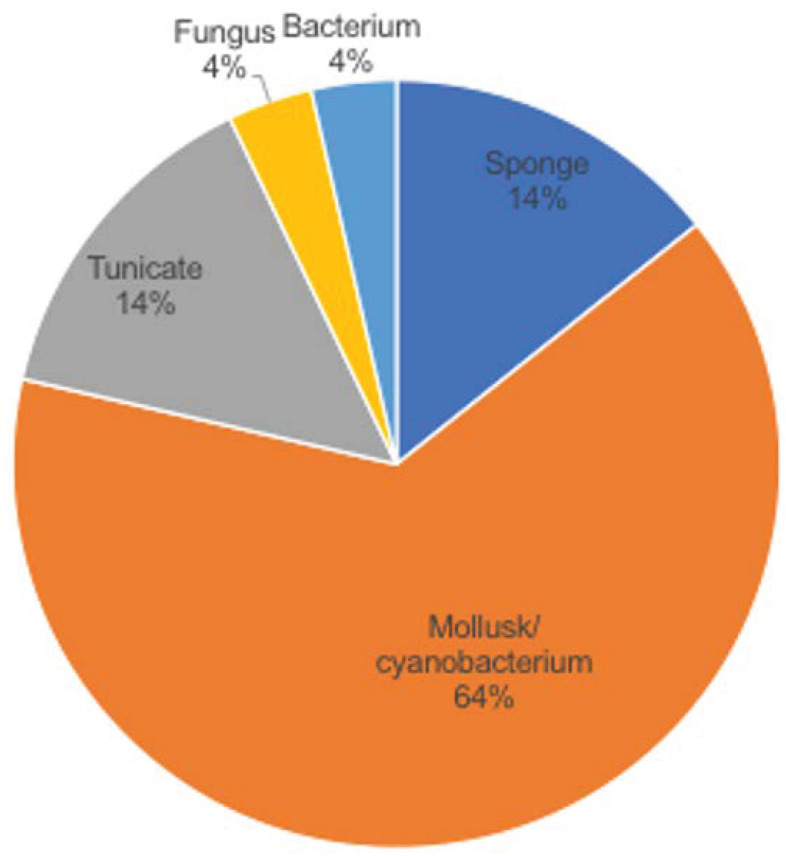
Distribution of resource organisms for commercial and clinical phase marine drugs.

Nature is a major resource of multiple chemical components, and most of them are natural products or derived from them. The ocean, accounting for around 70% of earth, contains many organisms, which makes it a valuable source of biological compounds. Lots of marine-derived compounds can be used in pharmaceutical and therapeutical research [6]. The function of marine compounds varies from antibacterial, antidiabetic, antiviral, antifungal and anti-inflammatory to anticancer. Many commercial marine-derived compounds have demonstrated anticancer capabilities [6,7]. According to marine pharmacology, there was a total of 14 marine-derived compounds available on the market as of October 2020, and 9 of them are used as anticancer drugs (Figure 1, Table 1) [8]. Moreover, there were 19 marine-derived anticancer compounds in different phases of clinical trials [8]. Furthermore, numerous articles have illustrated the in vitro or in vivo anticancer capabilities of marine-derived compounds. The anticancer compounds derived from marine compounds mainly come from mollusk/cyanobacterium, sponge, tunicate, bacterium and fungus (Figure 2). This review provides an overview of the marine-derived compounds with anticancer properties, both their applications and anticancer mechanisms, and novel analytical technologies.

## 2. Overview on the Marine-Derived Anticancer Compounds

### 2.1. Commercial Marine-Derived Drugs

Back in 1969, the first approved marine-derived compound, cytarabine (Ara-C, Cytosar-U), a synthetic pyrimidine nucleoside, was authorized by the United States Food and Drug Administration (FDA) to act as a first-line drug against leukemia [9]. Leukemia is a kind of blood cancer that is characterized by excessive unmatured leukocytes, which cause a lack of normal white blood cells and, thus, a series of symptoms, including bleeding, fatigue, infection or even death. According to *Global Cancer Statistics 2020*, an estimated 0.5 million new leukemia cases and about 0.3 million leukemia deaths happen worldwide [1]. After being administrated into the plasma, Ara-C is transported inside the cell by the human equilibrative nucleoside transporter 1 (hENT1). Once inside the cell, Ara-C is phosphorylated to its active form Ara-CTP, which further competes with deoxycytidine triphosphate (dCTP) as well as inhibits DNA polymerase α (DNA POL) activities, causing inhibition of DNA synthesis [10,11].

Then, in 2010, 41 years after Ara-C’s approval, the next commercial marine-derived drug, eribulin mesylate (Halaven), was approved by the FDA to treat metastatic breast cancer [9]. Eribulin mesylate is a synthetic derivative of marine product halichondrin B. It acts as a non-taxane microtubule-targeted drug. Through binding to tubulins and microtubules in the interphase, eribulin mesylate can suppress the centromere’s dynamics, arrest mitosis and, hence, cause proliferative inhibition as well as apoptosis of cancer cells [12,13].

One year later, after eribulin mesylate’s approval, brentuximab vedotin, the first antibody–drug conjugate (ADC) conjugating with marine product, was approved by the FDA for the treatment of systemic anaplastic large-cell lymphoma (ALCL) and Hodgkin lymphoma in 2011 [9]. Antibody–drug conjugates (ADCs) are the most thrilling of the oncology therapeutics emerging during these last few years. They are based on the principle of linking a cytotoxic drug to the monoclonal antibodies (mAbs), which can specifically target the antigen that overexpress on the surface of cancer cells. This strategy allows the cytotoxic drug to go inside the antigen-positive cancer cells and then cause apoptosis or inhibition without hurting normal cells [14]. Brentuximab vedotin is an ADC conjugating with CD30 mAb and a cytotoxic drug—monomethyl auristatin E (MMAE) by a linker [8]. CD30 is usually overexpressed on the surface of lymphoma cells. Moreover, monomethyl auristatin E (MMAE) is derived from auristatins and usually acts as the ADC payload. Auristatins are analogues of marine product dolastatin 10, which can inhibit tubulin polymerization, leading to arrest of the cell cycle and, ultimately, apoptosis of cancer cells. Unfortunately, due to their powerful cytotoxicity, auristatins need to be modified to be a tolerable drug. The most well-known compounds derived from auristatins are MMAE and monomethyl auristatin F (MMAF) [14]. Conjugating mAb targeting CD30 and cytotoxic MMAE empowers brentuximab vedotin with the ability to enter into the CD30-positive lymphoma cells and interfere with microtubule formation, thus causing cell cycle arrest and inducing apoptosis [14,15].

Unlike previous marine drugs, trabectedin (Yondelis), the natural alkaloid initially isolated from marine tunicate, exerts its anticancer functions through many different mechanisms [16]. Firstly, it can induce the break of DNA double strands by binding to the minor groove of DNA. Secondly, it can cause cell cycle arrest by disrupting microtubules and then interfering with late S and G2 phases. Moreover, it is also capable of inducing degradation of the RNA polymerase II (RNAPII). Furthermore, it can also modulate the tumor microenvironment by inhibiting the release of cytokines [17,18]. With its various anticancer mechanisms and potent effects, trabectedin was approved by the FDA to treat advanced soft tissue sarcoma and ovarian cancer in October 2015 [8].

The first marine product that went into the clinical trials for cancer therapy was didemnin B, a cyclic depsipeptide isolated from the Caribbean tunicate. However, it failed to pass the clinical trials because of its inefficiency and toxicity [31]. Then, plitidepsin, which is structurally similar to didemnin B but more powerful and less toxic, was isolated from the Mediterranean tunicate and was approved by the Australian regulatory authorities to treat myeloma, leukemia and lymphoma in December 2018 [8]. The main mechanism of plitidepsin was achieved through targeting eukaryotic elongation factor 1A2 (eEF1A2) and then causing apoptosis of cancer cells. The target of plitidepsin, eEF1A2, is one of the two isoforms of the protein elongation factor eukaryotic elongation factor 1 (eEF1A). As an elongation factor protein, eEF1A can mediate aminoacyl-tRNA recruitment to the ribosome during the translation. It shows pro-oncogenic activities and is usually overexpressed in many tumors, such as multiple myeloma, breast cancer and lung cancer [32]. Moreover, plitidepsin can also inhibit the cell cycle and cause apoptosis via G1 and G2/M arresting and sustained activation of the Rac1/JNK pathway [19,20,21]. Finally, plitidepsin was also found to induce proteotoxic apoptosis by generating endoplasmic reticulum stress and inhibiting autophagy [22].

As mentioned above, the discovery and approval of ADCs have paved the way for the development of more specific and effective therapeutic agents for cancer therapy. This led to a thrilling result, as the FDA authorized three more ADCs for oncotherapy in the last two years, namely, polatuzumab vedotin (Polivy, Pola), enfortumab vedotin (PADCEVTM) and belantamab mafodotin (Blenrep) [8].

Polatuzumab vedotin, approved by the FDA on 10 June 2019, was applied for the therapy of non-Hodgkin lymphoma (NHL), chronic lymphocytic leukemia (CLL), lymphoma, B-cell lymphoma and follicular lymphoma (FL) [8]. It is an anti-CD79b ADC linking with MMAE. CD79b, a component of the B-cell antigen receptor (BCR), is overexpressed on the surface of lymphoma B cells and causes proliferation by activating the immunoreceptor tyrosine-based activation motif (ITAM) and PI3 kinase (PI3K) signaling pathways [33]. After binding to the CD79b expressing on the B-cell surface, polatuzumab vedotin is internalized. Its linker will be cleaved, which will cause the release of MMAE inside the cell by division inhibition and apoptosis of the cell [23,24].

Enfortumab vedotin, an ADC designed to treat cancer-expressing nectin-4, was approved by the FDA to treat metastatic urothelial cancer in December 2019 [8]. Nectin-4 (PRR4) is a member of the nectin family that belongs to the immunoglobulin superfamily and acts like an adhesion molecule [34]. It is overexpressed on the surface of many epithelial cancers, including bladder cancer, lung cancer, breast cancer and pancreatic cancer, which makes it an attractive target for cancer therapy. Conjugating anti-nectin-4 antibody with the potent microtubule-disrupting agent MMAE offers enfortumab vedotin the ability to target the nectin-4-positive cells and then induce cell death through MMAE [25].

Belantamab mafodotin (belamaf), another ADC consisting of an anti-B-cell maturation antigen (BCMA) mAb and the active cytotoxic drug monomethyl auristatin F (MMAF), was approved for the therapy of relapsed/refractory multiple myeloma (RRMM) in August 2020 by the FDA [8]. The B-cell maturation antigen (BCMA) is one of the three receptors of the B-lymphocyte stimulator (BLyS). BLyS belongs to the TNF family and is critical in maintaining normal B-cell development and homeostasis [35]. BCMA is predominantly expressed on B lymphocytes, making it an effective therapeutic target of lymphoma. Monomethyl auristatin F (MMAF), structurally similar to MMAE, is also a tubulin polymerase inhibitor that can cause cell cycle arrest and apoptosis [36]. Integrated with the BCMA antibody and MMAF, belantamab mafodotin can specifically bind to BCMA-positive myeloma cells and eliminate them by inhibiting microtubule polymerization. In addition, it can also enhance the recruitment and activation of immune effector cells [26].

In the meantime, lurbinectedin (Zepzelca), a derivative of ecteinascidin (a marine agent isolated from the sea squirt species Ecteinascidia turbinate), was approved by the FDA to treat metastatic small-cell lung cancer (SCLC) in June 2020 [8]. Structurally similar to trabectedin, lurbinectedin can also covalently bind to the DNA promoter and lead to the break of DNA double strands, causing apoptosis of cancer cells [27,28]. Moreover, it can also inhibit the activity of RNA polymerase II and promote its degradation, which also leads to the DNA double-strand break of cancer cells [29]. Furthermore, it can also modify the tumor inflammatory microenvironment by inhibiting transcription of tumor-associated macrophages, enabling it to kill cancer cells comprehensively [30].

### 2.2. Marine-Derived Compounds in Phase III Clinical Status

According to marine pharmacology, there are four marine-derived compounds in phase III clinical trials, and two of them were used as anticancer drugs, which are marizomib (salinosporamide A; NPI-0052) and plinabulin (NPI-2358) (Table 2) [8].

Marizomib (salinosporamide A; NPI-0052), acting as an irreversible proteasome inhibitor derived from marine bacterium Salinispora tropica, is now in phase III clinical trials treating non-small-cell lung cancer (NSCLC), pancreatic cancer, melanoma, lymphoma and multiple myeloma [8]. Proteasome is a part of the ubiquitin–proteasome system (UPS), whose imbalance will cause a loss of cellular homeostasis and induce inflammation or cancer development [48]. Marizomib is a potent proteasome inhibitor that can inhibit all three proteolytic activities of the proteasomes slowly but irreversibly, resulting in the accumulation of abnormal proteins and finally introducing apoptosis of cancer cells [37]. In addition, marizomib is also capable of activating caspase apoptosis [38,39], decreasing the membrane potential of mitochondrial as well as increasing production of superoxide, making it a promising anticancer drug. [40]. In a phase I study of marizomib (NPI-0052) in patients with advanced malignancies, 86 patients were enrolled (solid tumors (including melanoma, colorectal cancer, stomach cancer and prostate cancer): *n* = 24; multiple myeloma: *n* = 35; lymphoma (including Hodgkin lymphoma and non-Hodgkin lymphoma): *n* = 22; leukemia (including chronic lymphocytic leukemia): *n* = 5). A total of 42 patients received treatment weekly (schedule A), while the other 44 patients received treatment twice weekly (schedule B). The recommended phase 2 doses (RP2D) from schedule A and schedule B were 0.7 mg/m^2^ over 10 min and 0.5 mg/m^2^ over 2 h, respectively. The most common related treatment emergent adverse events (TEAEs) observed in both schedules were fatigue, nausea, diarrhea and infusion site pain. One patient with transformed marginal zone lymphoma in schedule A had complete response. As for schedule B, the overall response (OR) rate was 11%, all of which were observed in 27 relapsed and/or refractory multiple myeloma (RRMM) patients [41]. Another phase I trial (NPI-0052-107) evaluating marizomib (0.3–0.5 mg/m^2^), pomalidomide (3–4 mg) and low-dose dexamethasone (0.5 mg/m^2^) was carried out in 38 RRMM patients. The RP2D determined by this trial was 0.5 mg/m^2^. The most common TEAEs were pneumonia, anemia, neutropenia and thrombocytopenia. The OR rate in this trial reached 53% (19/36), and the clinical benefit rate (CBR) reached 64% (23/36) [42]. Another phase I clinical trial assessing the effect of marizomib combined with the histone deacetylase inhibitor vorinostat was also conducted. A total of 22 patients participated in this clinical trial (17 with melanoma, 4 with pancreatic cancer and 1 with NSCLC). The outcome was very promising with a stable disease rate of 61%, and 39% of the participants’ tumor measurements decreased. Fatigue, anorexia, nausea, vomiting and diarrhea were the most common TEAEs that could be observed during this trial [43].

Plinabulin (NPI-2358), a synthetic analog of the marine fungus product phenylahistin, is now going through phase III clinical trials as a drug against non-small-cell lung cancer and brain tumors [8,44]. Plinabulin is a specific and potent anti-microtubule agent. It can induce apoptosis by inhibiting the polymerization of tubulin and activating caspase pathways [45]. Furthermore, compared to classic tubulin stabilizing drugs, plinabulin penetrates tissue more easily and is safer for cancer patients [45]. In a phase I study evaluating plinabulin’s effect in solid tumors and lymphoma, 38 patients were enrolled for the evaluation. Based on the outcome, 30 mg/m^2^ was selected as the RP2D dose. Adverse events observed in this trial included nausea, vomiting, fatigue and fever. Moreover, a total of 30% of stable disease was observed, among which four patients were able to maintain for more than 4 months [46]. In a phase II study of plinabulin with docetaxel, 172 patients with advanced NSCLC were tested. The OS rate of the combined group was 8.7%, while in the docetaxel group it was 7.5%, and the response rate was 14.0% and 14.5% in the combined group and the docetaxel group, respectively. The OS rate of patients with lung tumors over 3 cm was 11.5% in the combined group and 7.8% in the docetaxel group. Nausea, fatigue, anorexia, constipation and diarrhea were the most common side effects observed within this trial [47].

### 2.3. Marine-Derived Compounds in Phase II Clinical Status

Based on marine pharmacology, 12 marine compounds are in phase II clinical trials, among which 10 of them are tested as anticancer drugs (Table 3). To improve the specificity and potency of cancer drugs, ADCs are receiving growing interest, and more specific antigens are required for the development of novel ADCs. There is no surprise that 9 of the 10 marine-derived compounds in phase II studies are ADCs, as described below.

W0101 is a novel insulin-like growth factor type 1 receptor (IGF-1R)-targeting ADC, which was designed to deliver cytotoxic MMAE to the IGF-1R-overexpressing cancer cells [8,49]. The IGF-1R is a transmembrane tyrosine kinase that can induce cellular proliferation after activation. It is significantly overexpressed in various solid tumors, making it a marker of tumorigenesis and a promising target for therapeutic agents [77]. Consisting of the mAb specifically targeting IGF-1R and the cytotoxic MMAE, W0101 is capable of anti-proliferation and is now under phase II clinical trials to treat advanced or metastatic solid tumors [50]. In the IGF-1R 3+ MCF7 breast cancer model, treatment with 3 mg/kg W0101 was shown to cause 90% tumor growth inhibition (TGI) [49]. In another preclinical study of W0101, animal experiments of MCF-7 (breast cancer, IGF-1R 3+), CAOV3 (ovarian cancer, IGF-1R 2+), NCI-H2122 (lung cancer, IGF-1R 2+), SBC5 (lung cancer, IGF-1R 1+) and Hs746T (gastric cancer, IGF-1R−) were carried out. Except the 1+ lung cancer SBC5 and the gastric cancer IGF-1R-Hs746T animal models, all the other animal models observed potent tumor regression even in the docetaxel-resistant MCF-7 tumor model, showing that W0101 was an IGF-1R dependent and effective compound for cancer therapy [50].

CX-2029 (ABBV-2029), a special ADC against CD71 (transferrin receptor 1), also defined as a Probody drug conjugate (PDC), is now being tested in phase II clinical trials to treat solid tumors, head and neck cancer, non-small-cell lung cancer, pancreatic cancer and diffuse large B-cell lymphoma [8]. Unlike the direct targeting ability of ADCs, Probody drug conjugates (PDCs) link the cytotoxic drug with the protease-activated antibodies [78]. Proteases are usually upregulated in the tumor microenvironment (TME) since they are necessary for tumor growth, proliferation and metastasis. The protease-activated antibodies stay inactivated before entering the TME. Once encountering the protease around the tumor, the protease-activated antibodies of the PDCs are activated. The antibodies can then directly bind to the antigen expressing on the surface of cancer cells. After that, the conjugating cytotoxic drug is able to enter inside the tumor [78]. CD71, also known as transferrin receptor protein 1 (TfR1), is vital for the intake of transferrin-iron complexes and is widely expressed in normal cells yet highly expressed in cancer cells [79]. Conjugating with MMAE, CX-2029 is a promising and safe drug for cancer therapy [51,52]. CX-2029 was shown to be well tolerated and inhibit tumor growth in multiple solid tumor and lymphoma animal models as well as in NSCLC models and diffuse large B-cell lymphoma (DLBCL) animal models [52,53]. Moreover, a phase I study in patients with advanced cancers generated a dose of 3 mg/kg CX-2029 for phase II clinical trials, and anticancer activity in head and neck as well as non-small-cell lung cancers was observed when the dose was equal to or higher than 2 mg/kg. Adverse effects included anemia, neutropenia and thrombocytopenia [51].

CAB-ROR2 (BA-3021) is an ADC targeting receptor tyrosine kinase-like orphan receptor 2 (ROR2) using the novel conditionally active biologics (CAB) technology [8,54]. CAB technology is a novel technology that allows the antibodies to selectively bind to the target antigen of the cancer cells instead of the normal cells, which is based on their unique TME, such as energy metabolism (including the Warburg effect) [80]. ROR2 is a receptor tyrosine kinase orphan receptor (ROR) family member for the Wnt signaling pathways. Not only highly expressed in many various tumors, ROR2 is also expressed in a wide range of normal tissues [81]. Using the CAB technology, CAB-ROR2 (BA-3021), linking to MMAE, could be specifically activated by the glycolytic metabolism of TME without affecting the normal tissues [54]. BA3021 was able to decrease the growth of human melanoma tumor (SK-MEL-5) xenografts and sarcoma cancer patient-derived xenograft models [54]. It is now under phase II clinical trials for the therapy of solid tumors, non-small-cell lung cancer, triple-negative breast cancer and soft tissue sarcoma [8].

RC-48, an ADC consisting of MMAE and the mAb targeting human epidermal growth factor receptor 2 (HER2), is currently under phase II clinical trial to treat urothelial carcinoma, advanced cancer, gastric cancer, HER2-overexpressing gastric carcinoma, advanced breast cancer and solid tumors [8,55]. HER2 is a member of the human epidermal growth factor receptor (HER) family and is widely as well as highly expressed in multiple solid cancers [82]. Conjugating with the anti-HER2 antibody and MMAE, RC-48 can target the HER2-positive cancer cells and disrupt cancer cells by MMAE’s cytotoxicity [56]. In a phase I trial evaluating the effect of RC48 in HER2-overexpressing advanced or metastatic solid carcinoma patients (especially gastric cancer), RC48 was well tolerated and displayed encouraging antitumor activity in HER2-positive solid tumors with a 21.0% (12/57) objective response rate (ORR) and 49.1% (28/57) disease control rate (DCR). The most common TRAEs were hypoesthesia, leukopenia, neutropenia and increased conjugated blood bilirubin [57]. In its phase II study carried out in 43 locally advanced or metastatic HER2+ urothelial carcinoma patients, RC48 was promising in anti HER2+ cancer with a 51.2% ORR, a median 6.9 months of progression-free survival (PFS) and 13.9 months of overall survival (OS). Leukopenia, alopecia and hypoesthesia were the most frequent TEAEs [56].

Enapotamab vedotin (HuMax-AXL) is a novel ADC conjugating with a human AXL-specific IgG1 and MMAE, and it is now under the test of phase II clinical trials for the treatment of ovarian cancer, cervical cancer and endometrial cancer [8,58]. AXL is a member of the receptor tyrosine kinase family (RTKs), relating to the proliferation and invasion of cancer cells. Overexpression of AXL has been reported in a multitude of tumors, which makes it an attractive target for cancer therapy [83]. Linking the AXL-specific IgG1 and MMAE enables enapotamab vedotin to target the AXL-positive cancer cells and cause inhibition via MMAE [58]. A phase I trial assessing enapotamab vedotin in 46 patients carrying solid tumors (8 with NSCLC, 9 with melanoma, 22 with ovarian, 3 with cervical and 5 with endometrial cancer) was conducted, leading to a 2.2 mg/kg dose of RP2D. Three patients (two NSCLC with 2.2 mg/kg dose treatment and two ovarian with a dose of 1.5–2.4 mg/kg treatment) showed partial response. Common side effects included diarrhea, vomiting, constipation, fatigue and nausea [59]. In a phase 2a trial of stage III/IV NSCLC patients, enapotamab vedotin monotherapy was proved to be safe with a dose of 2.2 mg/kg. Moreover, the clinical activity of enapotamab vedotin was also promising with an ORR of 19% and a disease control rate (CR+PR+SD) of 50% (13/26). Adverse events contained nausea, vomiting, diarrhea and colitis [60].

Telisotuzumab vedotin (ABBV-399), the ADC consisting of the c-Met antibody ABT-700 and MMAE, is designed to treat c-Met-amplified solid tumors and is now under phase II clinical status [8,61]. C-Met is a receptor tyrosine kinase (RTK) family member, acting as the receptor for hepatocyte growth factor (HGF) and causing tumor genesis. C-Met is highly expressed on the surface of various solid tumors, making it a therapeutic spot for malignancy [84]. Consisting of c-Met antibody ABT-700 and the tubulin inhibitor MMAE, telisotuzumab vedotin is able to kill the c-Met overexpressing cancer cells via MMAE [61]. In phase I studies evaluating telisotuzumab vedotin’s effects in 48 patients with advanced solid tumors (17 with non-small-cell lung cancer and 16 of them were c-Met positive NSCLC, 12 with nonsquamous, 5 with squamous, 4 with breast cancer, 9 with colon/rectal cancer, 2 with endometrial cancer, 4 with ovarian cancer and 12 with other solid cancers), the recommended dose of phase II was 2.7 mg/kg. Among the 16 patients with c-Met-positive NSCLC, three patients (18.8%) achieved PR and two patients showed a significant reduction in lesions. However, no other participants observed had a response to telisotuzumab vedotin monotherapy. The most frequent telisotuzumab vedotin-related adverse events were fatigue, anemia, neutropenia and hypoalbuminemia (4% each) [62]. Another phase I study of telisotuzumab vedotin monotherapy in patients with advanced solid tumors was carried out in Japan. Nine patients with solid tumors were enrolled, including NSCLC (*n* = 2), esophageal cancer (*n* = 1), thymic cancer (*n* = 1), breast cancer (n = 1), pancreatic cancer (*n* = 1), ovarian cancer (*n* = 1), urothelial carcinoma (*n* = 1) and liposarcoma (*n* = 1). Telisotuzumab vedotin was well tolerated at a dose of 2.7 mg/kg in this trial. The most common TEAEs were peripheral decreased appetite, nausea, sensory neuropathy and decreased white blood cell count. For the clinical activity, six (67%) had stable disease, two patients (22%) achieved a PR (both were c-Met positive, one with urothelial cancer and the other with ovarian cancer) and one (11%) had progressive disease [63]. In a phase II study of telisotuzumab vedotin in c-Met-positive stage IV or recurrent squamous cell lung cancer patients, 28 patients were enrolled. At the end, two responses (9%) were reported and 10 patients had stable disease. The most common side effects were fatigue, pneumonitis and hypophosphatemia [64].

Ladiratuzumab vedotin (SGNLIV1A), an ADC composed of a humanized anti-LIV-1 antibody coupled with the MMAE, is currently under phase II investigation of breast cancer [8,65]. LIV-1 is a transmembrane protein belonging to the subfamily of zinc transporters. It plays an important role in cancer growth and metastasis and highly expresses in multiple solid cancers, including breast cancer [85]. After binding to the HIV-1 positive breast cancer cells, the tubulin inside the cancer cell is disrupted by MMAE [65]. Moreover, ladiratuzumab vedotin can elicit immunogenic cell death (ICD) through the induction of endoplasmic reticulum stress [66]. In a phase Ib/II study of the combination of ladiratuzumab vedotin and pembrolizumab in triple-negative breast cancer patients, 51 patients were enrolled. In this ongoing trial, 26 patients were assessed, and the confirmed OR rate was 54%. The most common TEAEs were diarrhea, fatigue, hypokalemia, alopecia nausea and constipation [67].

Tisotumab vedotin (TV), an ADC composed of tissue factor (TF)-directed mAb and tubulin inhibitor MMAE, is now in phase II investigations for solid cancers [8,68]. The tissue factor (TF) is considered to be a factor that initiates thrombin formation from the zymogen prothrombin and causes blood coagulation. Except for its physiological clotting role, TF is also found to cause tumor angiogenesis and metastasis and is aberrantly expressed on many solid cancers, such as cervical cancer, ovary cancer, bladder cancer and lung cancer. Downregulation of TF is capable of inducing apoptosis and impairs cell survival of tumor cells, leading it to be a potential target for cancer therapy [86]. Consisting of TF-directed mAbs and MMAE, tisotumab vedotin can induce cytotoxicity via the MMAE mechanism. In addition, tisotumab vedotin can also induce ICD and bystander cytotoxicity of cancer cells [68]. In its phase I and phase II clinical studies, in order to evaluate the effect of tisotumab vedotin in patients with advanced or metastatic solid tumors (including bladder, cervix, endometrium, esophagus, NSCLC, ovary, prostate and squamous cell carcinoma of the head and neck (SCCHN)), 27 patients were enrolled for phase I dose escalation and 147 patients were enrolled for phase II dose expansion. In 2015, a recommended dose of 2.0 mg/kg of tisotumab vedotin was given by the phase I study. In its phase II study, a 15.6% (23/147) OR rate was observed across all the tumor types, which consisted of 26.7% (4/15) in bladder cancer, 26.5% (9/34) in cervical cancer, 7.1% (1/14) in endometrial cancer, 13.3% (2/15) in esophageal cancer, 13.3% (2/15) in NSCLC, 13.9% (5/36) in ovarian cancer and 0% (0/18) in prostate cancer. The common side effects observed in the phase II study were alopecia, epistaxis, dry eye, fatigue, conjunctivitis, nausea, decreased appetite and vomiting [69].

AGS-16C3F, an ADC targeting ectonucleotide pyrophosphatase/phosphodiesterase 3 (ENPP3, CD203a) conjugated to MMAF, is now a subject of phase II clinical trials for the therapy of renal cell carcinoma (RCC) [8,70]. ENPP3 (CD203a) is an ectoenzyme that is involved in ATP pyrophosphatase activities and hydrolysis of extracellular nucleotides. It was reported to promote invasion and metastasis of cancer cells and is abundantly expressed in the human cyclic endometrium and many solid tumors, including RCC [87]. AGS-16C3F is a novel ADC against ENPP3-positive RCC, causing cell cycle arrest and apoptosis with its conjugation cytotoxic MMAF [70]. In a phase I study of GS-16M8F and AGS-16C3F in advanced refractory renal cell carcinomas (RRCC), a recommended dose of AGS-16C3F for further phase II clinical trials was set to be 1.8 mg/kg. In addition, antitumor activity was also observed (23% of PR (3/13), 92% of disease control rate (12/13)) [71]. However, in its phase II study combined with axitinib in 84 previously treated metastatic renal cell carcinoma patients (mRCC), treatment with AGS-16C3F failed to meet its primary and secondary endpoint, even though it was proved to be safe at a dose of 1.8 mg/kg [72].

Plocabulin (PM184), originally isolated from the marine sponge, is a polyketide acting as a new tubulin-binding agent and is now under phase II clinical tests [8,73]. Plocabulin targets the tubulin dimers at a new binding site and causes apoptosis by inhibiting tubulin polymerization [73,74]. Moreover, plocabulin is also reported to inhibit angiogenesis in endothelial cells [75]. In a phase I study of plocabulin in patients with advanced solid tumors, 44 candidates were treated and evaluated (11 with colorectal adenocarcinoma, 5 with breast carcinoma, 5 with cervix carcinoma, 5 with NSCLC, 3 with a gastrointestinal stromal tumor, 3 with pancreas adenocarcinoma, 3 with soft tissue sarcoma and 9 with other cancers). The recommended dose for phase II was not determined in this study. However, the anticancer activity was promising with a clinical benefit of 33%, and the common side effects included diarrhea, alopecia, fatigue, anorexia and myalgia [76].

### 2.4. Marine-Derived Compounds in Phase I Clinical Status

In line with marine pharmacology, there are seven marine compounds in phase I clinical status, and six of them are used as anticancer drugs (Table 4).

MORAb-202 is an ADC consisting of the antibody farletuzumab and the microtubule inhibitor eribulin, and it is currently under phase I clinical investigation for solid tumors [8,88]. The antibody farletuzumab is designed to target the human folate receptor alpha (FRα), a folate-binding protein belonging to the folate receptor (FOLR) family. It is excessively expressed on the surface of many solid tumors, such as ovarian cancers, breast cancers and lung cancers, making it an exciting candidate for cancer therapy [105]. The microtubule inhibitor, eribulin, is a derivative of marine product halichondrin B and demonstrates potent antitumor activity via inhibiting microtubule’s elongation [88]. In possession of both the anti-FRα antibody and the microtubule inhibitor eribulin, it allows MORAb-202 to become a potent as well as specific cytotoxic drug for FRα-positive cancer cells [88]. In a phase I study of MORAb-202 in patients with folate receptor-α-positive advanced solid tumors, 22 patients were enrolled (cancer types included ovarian, breast, endometrial, NSCLC and fallopian tube cancer). The maximum tolerated dose of MORAb-202 was not reached, and the adverse events included leukopenia and neutropenia. A promising anticancer activity was observed (one complete response (CR), nine partial response (PR) and eight stable disease) within this clinical trial [89].

XMT-1536 is another novel ADC consisting of anti-NaPi2b antibody and an auristatin derivative and is currently under phase I clinical evaluation to treat solid tumors [8,90,91]. After entering the NaPi2b-positive cancer cells, XMT-1536 can release the cytotoxic auristatin derivative to kill the cancer cells via microtubule inhibition and the bystander effect [91,92]. NaPi2b is a type II sodium-dependent phosphate transporter encoded by the SLC34A2 gene. NaPi2b is involved in maintaining homeostasis and is highly expressed on the surface of ovarian cancer and lung cancer, leading it to be a novel candidate for cancer targeted therapy [106]. In a phase I study of XMT-1536, 36 patients with solid tumors expressed NaPi2b (22 with ovarian, 7 with endometrial, 4 with NSCLC and 3 with other cancers). XMT-1536 was shown to be well tolerated up to 30 mg/m^2^. The most common adverse events contained nausea, anorexia, vomiting, headache, fatigue and myalgia. In total, 2 PR and 11 SD were observed, which illustrated the anticancer activity of XMT-1536 [93]. In another phase I study of XMT-1536 in 23 pretreated metastatic ovarian cancer (OC) and NSCLC (19 with OC and 4 with NSCLC), XMT-1536 was also well tolerated and demonstrated anticancer activity in OC and NSCLC adenocarcinomas. The common adverse events included nausea, fatigue and pyrexia [94].

Among the phase I clinical marine compounds, six of them were designed for anti-HER2-positive cancers, which were PF-06804103, ARX-788, ALT-P7 and ZW-49.

PF-06804103, an ADC conjugating the antibody targeting HER2 and the MMAE variant Aur-101, is in phase I clinical status as an anticancer drug treating breast neoplasms, stomach neoplasms, esophagogastric junction neoplasms, carcinoma and non-small-cell lung cancer [8,95]. PF-06804103 was reported to be safer and more effective than the common HER2-targeted drug trastuzumab with its anti-tubulin effect, impaired lysosomal degradation and outstanding bystander effect [96,97]. In a phase I study of PF-06804103 in patients with HER2-positive advanced breast cancer (BC) or gastric cancer (GC), 35 patients were enrolled (20 with BC and 15 with GC). Common adverse events included fatigue, alopecia, arthralgia, myalgia, neuropathy and osteomuscular pain. It also showed an encouraging anticancer outcome with an ORR of 52.4% (11/21) in the dose ≥3mg/kg in patients [98].

ARX-788 is another HER2-targeted ADC combined with MMAF [99,100], while ALT-P7 [102] as well as ZW-49 [104] are both conjugated with MMAE, but all of these were under investigation for the treatment of breast cancer and/or gastric cancer [8].

In a phase I study of ARX788 in patients with metastatic HER2-positive breast cancer, 45 patients were enrolled. ARX788 was well tolerated, and 1.3 mg/kg was the recommended dose for the phase II study. It also illustrated anticancer capability with an OR rate of 31% (13/42) in the evaluation of participants and 42% (5/12) in the 1.3 mg/kg therapy [101]. In the phase I study of ALT-P7 in patients with HER2-positive advanced breast cancer, 27 patients were enrolled. ALT-P7 was well tolerated at 4.5 mg/kg. The common adverse events included neutropenia, pruritusand, fatigue, myalgia, sensory neuropathy and neutropenia. The disease control rate was 77.3% (17/22) among the evaluated patients, and the PR rate reached 13.3% (2/15) in patients with measurable lesions, which was a good indication for the phase III study [103]. In in vivo experiments, ZW-49 was shown to inhibit breast cancer growth both in cancer cell lines and breast cancer xenograft (PDX) tumor models expressing HER2 (low and high HER2 expressing) [104].

### 2.5. Potential Marine-Derived Anticancer Drugs

Apart from the commercial and clinical phases of drugs, numerous marine-derived compounds act as potential anticancer drugs among various cancer cells or animals.

The batzellines obtained from Caribbean sponge [107], extracts from marine sponge [108] and the epidithiodiketopiperazine DC1149B isolated from marine Trichoderma’s extraction [109] were reported to inhibit pancreatic carcinoma. The Dictyota dichotoma (Phaeophyceae) from marine macroalgae [110], the compound akiyoshiensis GRG 6 (KY457710) from marine Streptomyces [111] along with coibamide A (CA) from marine cyanobacterium [112] were able to cause apoptosis in breast cancer cells. The chromomycin SA analogs isolated from marine-derived Streptomyces [113], the cyclic lipoheptapeptides isolated from marine algicolous bacterial [114] and three chromone derivatives isolated from marine-derived Penicillium citrinum [115] were shown to work against lung cancers. The extracts derived from marine sponges [116], alkaloid aaptamine from marine sponges [117] and some extracts from marine fungus [118] decreased the proliferation of liver cancer cells. The salarin C extracted from sponge [119] and yessotoxins (YTXs) produced by marine dinoflagellates [120] were able to kill leukemia cells. Three compounds from marine invertebrates were indicated to cause apoptosis of glioblastoma cancer [121]. Neoechinulin A, isolated from marine fungus, has shown the cytotoxic effect on cervical cancers [122]. Ilimaquinone and ethylsmenoquinone from marine sponge exhibited anti-colon cancer activity [123]. Marine algal compounds RU017 and RU018 were able to inhibit cancer stem cells (CSC) [124].

In addition, lots of marine-derived compounds have widely shown anticancer abilities, such as philinopside A isolated from the sea cucumber [125], a copper coordination compound ZZF51 isolated from a marine fungus [126], leucettamol A from the marine sponge [127], pyrroloazepinone and indoloazepinone from marine natural products [128], bastadins-6, -9 and -16 isolated from the marine sponge [129], α-pyrone derivatives from marine actinomycete Streptomyces [130], fascaplysin from marine sponges [131], extracts of two different starfish species [132], leucettamine B from marine sponge [133], crude venom from jellyfish [134] and lamellarin D and its derivatives from marine products [135].

## 3. Developing Technologies in Marine Drug Discovery

With social progress and rapid development, the requirement for a better physical condition is becoming an cutting edge issue. Numerous drugs were discovered and applied to improve the quality and quantity of healthiness. The ocean occupies about three-quarters of the earth and contains various organisms, plants and microorganisms, yet it is the source of much less drug products than those of the terrestrial kind. However, it has great potential for the discovery of novel drugs with its incomparable area and ecosystem. In the last decades, the development of science and technology has brought us into a new era with an extraordinary speed. Many of these technologies have also been used in the search for marine bioactive compounds and undoubtedly offer promise for their further discovery in the future.

**Figure 3:**
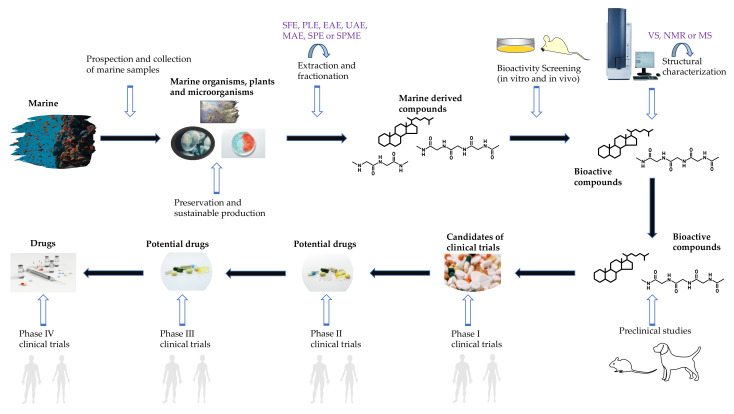
General procedures for marine-derived drug discovery.

The typical procedure for bioactive compound discovery contains a series of pivotal steps, including sample prospection, collection, preservation, extraction, fractionation, separation, purification, characterization and identification (Figure 3) [136]. Among these steps, sample extraction, separation, structural characterization and bioactivity identification are considered the most crucial steps since they significantly influence the quality and bioactivity of the anticipated compound (Table 5).

### 3.1. Emerging Technologies for Extraction and Separation

Traditionally, marine compounds were mainly extracted from the sample via solvent extraction and Soxhlet extraction with organic solvents, such as ethanol, chloroform and benzene, which is time consuming, resource wasting, low yielding and environment hurting. In order to improve the efficiency, many extracting strategies were invented and developed over the last decade, such as supercritical fluid extraction (SFE), pressurized liquid extraction (PLE), enzyme-assisted extraction (EAE), ultrasound-assisted extraction (UAE), microwave-assisted extraction (MAE), solid phase extraction (SPE) and solid phase microextraction (SPME) [136].

Supercritical fluid extraction (SFE) is a green extracting technique based on the supercritical fluids that are mainly generated by CO_2_. The supercritical fluid can evidently increase the sample’s dissolution via its potent diffusion inside the sample. Compared to traditional solvent extraction methods, SFE provides a much more efficient and economic method for isolating compounds from marine organisms [137]. In addition to being generated by pure CO_2_, the supercritical fluid generator could also be generated by mixed solvents. To get the best yield of the objective compound in a shorter time, temperature, pressure and solvent ratio of the reaction can be modified according to the compound’s features. In the extraction of chlorophyll compounds using SFE, the highest yield was found in the 70% cosolvent group, disregarding the cosolvent types [138]. SFE was also introduced to extract bioactive Tyrian purple precursors from marine gastropod and was shown to be safer than the traditional chloroform extraction [139]. Using SFE to extract pre-treating lipids from the marine diatom, a total of 27 fatty acids were extracted and identified, indicating SFE as an effective and harmless method for extracting compounds from marine organisms [140].

Pressurized liquid extraction (PLE) is one of the most promising extracting techniques with high pressure (50–300 psi) and high temperature (50–200 °C), mainly used in the extraction of solid or semi-solid materials. The high pressure and high temperature enable effective penetration and solubility of the solutes, which will undoubtedly improve the extraction efficiency and lower solvent and time consumption [141]. To achieve the most specific and efficient outcome, the operating conditions, including reaction time, pressure and temperature, should be carefully adjusted based on the properties of the desired product. In an experiment using the PLE to obtain the antioxidant protein from sea bass (muscle, head, viscera, skin and tailfin), the optimal conditions for different organs were diverse, pH 7 for muscle, viscera, skin and tailfins; pH 4 for head; 60 °C for head and tailfins; 55 °C for skin; 50 °C for viscera; 20 °C for muscle; 15 min for head, viscera and tailfins; and 5 min for muscle and skin [142]. Moreover, PLE can be connected with other analysis systems, such as the liquid chromatography–diode array detector–electrospray ionization mass spectrometry (LC–DAD–ESI/MS) system, leading it to be a synthetic analyzing system incorporating extraction, identification and quantification [143].

Enzyme-assisted extraction (EAE) is not actually a new extraction method. However, with optimization, combination with other extracting technologies and the arising of novel enzymes it is still considered an emerging technology. Two crude bacterial enzyme solutions were introduced to extract phycoerythrin (PE) and phycocyanin (PC) from Porphyra without limiting their bioactivity [144]. EAE was also found to be able to release specific bioactive tailor-made seaweed extracts via five carbohydrases and three proteases [145]. Using cellulase obviously enhanced brown seaweed and red seaweed protein products with bioactive functions [146]. In addition, coupling EAE with other techniques can dramatically increase the extraction yield and decrease extracting time without changing their features. A higher yield and shorter reaction times were detected when coupling the EAE and microwave-assisted extraction (MAE) methods to extract phenolic alcohols and acids from olive pomace (OP) [147] and to extract hypericin from hypericum [148].

Ultrasound-assisted extraction (UAE) is another efficient extraction method for bioactive compounds based on the cavitation of ultrasonic waves. The ultrasonic waves provide a stronger penetration of the solvent and straightforward disruption of cell membranes. Beyond that, it is also inexpensive, effective and maneuverable compared to orthodox methods. Many elements can affect the efficacy of UAE, such as ultrasound power, energy, frequency and working temperature [149]. In the extraction of phycobiliproteins from marine macroalgae, the best yield with UAE was observed when the ultrasonication amplitude maintained 120 µm for 10 min at 30 °C. When in combination with conventional methods, such as maceration and homogenization, UAE can significantly enhance the extraction efficiency, especially when combined with maceration [150].

Microwave-assisted Extraction (MAE) is a time- and solvent-saving extraction technology due to its physical mechanism [151]. Through microwave absorption, heat is generated within the whole material, causing entire dilapidation and, thus, the release of the molecules into the solvent [152]. To gain the best yield, several parameters can be modified based on the property of the sample, for instance, time, pressure or solvent ratio. In the extraction of plumieride from flower extracts, MAE produced almost twice or triplicate that of conventional methods under its optimal conditions (10 min, 300W) [153]. MAE could also be combined with other methods to enhance its productiveness. A MAE–DLLME (liquid–liquid microextraction)-GC/MS method was developed to extract and analyze 16 polycyclic aromatic hydrocarbons (PAHs) in smoked fish, which was proved to be a more accurate, rapid and reliable method [154].

Solid phase microextraction (SPME) is a solvent-free technology that combines extraction, isolation and concentration into one step, significantly reducing time and solvent. It involves the use of a fiber connected with an extracting phase and is based on the partition equilibrium of the extractives between their stationary phases. To gain the best output, many elements, such as extracting time, temperature and solvent volume, should be adjusted according to the properties of the different extractives. For example, in the extraction of parabens from lake water, SPME gave productivity of 70–98% under its optimized conditions (100 mL sample volume, 60 min for extraction, PH 8) [155]. To broaden its application, novel methods were introduced. In 2019, an on-fiber standard calibration method was developed to apply SPME to a semi-solid sample, which was shown to be practical, efficient and economical [156]. SPME could also be coupled with other methods, such as other technologies. An integrated SPME and GC/MS method was developed for extraction and determination of four biogenic amines in fish samples and was successfully applied to the samples with a recovery ranging from 78.9–110% [157].

Solid phase extraction (SPE) is an extracting method using a solid phase to absorb the desired compounds from the original sample. With an optimized setting, SPE can easily and automatically gain accurate compounds with less cost. Over the last decades, many new materials and methods were introduced for SPE. For instance, a novel carbonic material graphene was introduced to extract toxins from marine shellfish muscles with SPE, which was proved to be more effective and economic when compared with other common and commercial sorbents [158]. SPE could also be coupled with other technologies, such as MS, to make extraction and identification into one or two simple steps. An automated on-line SPE was coupled to LC-MS/MS to extract and determine the lipophilic toxins in marine shellfish, and several lipophilic toxins were found using this method, proving it to be a simple, rapid and cost reducing method [159].

Although the development or optimization of the extraction methods mentioned above have not yet occurred in the extraction of marine compounds, these emerging methods can be expected to undoubtedly promote the discovery of marine bioactive compounds.

### 3.2. Developing Technologies for Structure Characterization

After being extracted from marine samples, the mixture can then be subjected to different technologies that identify structures, such as vibrational spectroscopy (VS), nuclear magnetic resonance spectroscopy (NMR) or mass spectrometry (MS), to illustrate their structure [136].

Vibrational spectroscopy (VS) identifies the structure via the vibration generated by the absorption or emission of electromagnetic radiation. The two popular VS techniques are infrared (IR) spectroscopy and Raman spectroscopy. Infrared (IR) spectroscopy is a measurement spectroscopy via the absorption phenomenon of the infrared region within the electromagnetic spectrum. It can be used to identify chemicals and functional groups in the solid, liquid or gaseous phase. Unlike the absorption phenomenon of IR spectroscopy, Raman spectroscopy discriminates the structures through a nonelastic scattering of monochromatic light. It provides not only the information of chemical structures and functional groups but also their electronic states. The Fourier transform infrared (FT-IR) spectroscopy was introduced lately to determine the presence, type and quantity of polyhydroxyalkanoates without any prior purification [160]. In addition, FT-IR spectroscopy was also shown to be able to identify the subpopulations of extracellular vesicles from different sizes and different cellular origin efficiently and quickly [161]. Recently, Raman spectroscopy was indicated to identify and quantify the molecular modifications of collagen and seems to be an interesting tool to study biological processes [162]. A novel detecting method, single-cell Raman spectroscopy (scRS) coupled with one-dimensional convolutional neural networks (1DCNN) was explored to identify individual marine microorganisms quickly and accurately [163]. Moreover, combined microscopy–infrared (AFM-IR) spectroscopy and tip-enhanced Raman spectroscopy (TERS) provided a novel and automated approach to identify the structure of viruses [164].

Nuclear magnetic resonance spectroscopy (NMR) is a spectroscopic analytical technique for structure elucidation based on observing the magnetic properties around the atomic nuclei [165]. To obtain more effective and accurate identification, scientists have made an excellent effort towards NMR’s development, including its methodology and applications. For example, high-pressure NMR was introduced to measure molecules under variable pressure. Isotope-aided NMR allowed the automated and more accurate determination of larger proteins. The advanced magic-angle spinning (MAS) technology has enhanced the resolution and sensitivity of NMR [166]. For marine toxins, a pulse length-based concentration determination (PULCON) quantitative NMR (qNMR) with an external standard was introduced and successfully quantified them, which was shown to be a useful tool for the quantification of invaluable marine toxins [167]. Moreover, the benchtop NMR spectroscopy was recently illustrated to characterize the enzymatic hydrolysis reaction in real-time on red cod, salmon and shrimp [168].

Another analytical technology, mass spectrometry (MS), is an excellent and popular technique for structural identification. It can define the elemental or isotopic signature of a sample via its specific mass spectrum (mass-to-charge ratio of ions). Advanced MS-based methodologies have been developed by researchers for more accurate elucidation and broader application, such as matrix-assisted laser desorption/ionization (MALDI), electron impact/chemical ionization (EI/CI) and stable isotope labeling by amino acids (SILAC) [169]. In 2017, the matrix-assisted laser desorption ionization mass spectrometry in the TOF or TOF/TOF mode (MALDI-TOF MS) was discovered as an integrated procedure to identify and analyze the protein protease inhibitors from marine invertebrate extracts, which was considered to be a fast and sensitive approach for the discovery of proteinaceous ligands [170]. Furthermore, coupling MS with other technologies was testified to be a very common yet very powerful analyzing strategy. For example, coupling gas chromatography with triple quadrupole MS (GC–QQQ-MS) was shown to be a powerful tool to identify steryl glycosides in various marine microalgae [171]. Coupling HPLC to a quadrupole time-of-flight MS with the positive/negative electrospray ionization source has also been shown to successfully elucidate the structure of a new palytoxin congener isolated from the marine dinoflagellate, whereas at the same time, NMR gave limited information [172]. A synthetic method coupled with the liquid–liquid extraction, tandem liquid chromatography separations, and triple quadrupole time-of-flight MS/MS was used to efficiently separate and analyze marine toxins [173]. A comprehensive system consisting of electrospray ionization (ESI), hydrophilic interaction liquid chromatography (HILIC) and MS (sequential MSn (*n* = 2, 3) or Fourier-transform MS) was discovered and has successfully been applied to identify 22 arsenosugar phospholipids (As-PL) in marine algae [174]. A novel method based on ultra-high performance liquid chromatography (UHPLC)-MS was introduced to screen enzyme inhibitors on marine natural products, which was fast, convenient and sensitive compared to conventional inhibition assays [175].

With all the thrilling and emerging developments, marine compounds can be identified much more efficiently, and novel compounds can be discovered much more quickly.

### 3.3. Innovative Screening Methods for Bioactive Compounds

Marine extracts are an important and valuable source for the discovery of novel bioactive compounds. Countless people are suffering, owing to diverse illness. Thus, searching for bioactive compounds to fight against diseases is of great significance. However, screening and illustrating the bioactive compound is a great challenge for researchers. Classical bioactivity screening contains primary screens and secondary screens, and it includes both in vitro and in vivo assays to test the antimicrobial, anticancer, antiviral, anti-inflammatory and analgesic activities [183]. It is economically costly and time consuming to obtain a bioactive agent via the traditional screening method. Alternatively, the innovative screening method has speeded up the discovery and validation of bioactive compounds tremendously. Some representative novel screening methods are mentioned below.

In 2007, a high-throughput toxicity screen version, an automatic yeast model system, was discovered that analyzes multiple antimicrobial compounds at the same time [176]. Firstly, a 2× YPD-H (YPD media buffered with HEPES) solution was prepared, and the pH was adjusted to 7. After that, the 2× YPD-H solution was mixed with 2× agar to form a YPD-H-agar, which was inoculated with the yeast culture and then irrigated into an OmniTray. At the same time, the tested samples were prepared in a 384-well tray. Then, the samples were added into the YPD-H-agar via the automatic robot. Once the samples were added, the absorbance was read at 544 nm, and the absorbance was read again after 24 h incubation [176]. This novel screening was a quantitative method, and its efficacy was testified by over 3000 compounds, and it was proved to be a potent and speedy screening method for the identification of antimicrobial compounds from natural extracts.

To build the distinct biological fingerprints for antibiotics, an antibiotic mode of action profile (BioMAP) screening was established in 2012 [177]. The major steps consisted of the following: (1) plate the pathogenic strains into 384-well plates; (2) pin the compounds into each well via a pinning robot; and (3) stack the screening plates into an automated reader/shaker and then read the OD600 every hour, lasting for 24 h. This BioMAP screening method was verified by available commercial drugs and some known antibiotic extracts from certain microorganisms, proving itself an accurate and efficient tool to profile leading antibiotic compounds and predict novel antibiotics [177].

The advancement of omics, such as genomics, transcriptomics, proteomics and metabolomics, has enabled faster characterization and discovery of bioactive molecules. Large-scaled, multi-omics analyses of various compounds via different databases have provided a new powerful tool for drug development. In 2010, in an experiment looking for functional bioactive antinematode compounds from marine bacterium, the genomic library screening method was introduced and had increased the bioactivity screening efficiency dramatically [178]. The combination of the transcriptome outcome and proteomic results has led to the discovery of 238 novel peptides from scorpion venom, among which a new peptide showed the potential to inhibit cancer growth [179]. Recently, a computer-driven approach was introduced to discover natural products against methicillin-resistant Staphylococcus aureus (MRSA) infection [180]. This approach consisted of extracting molecules from different online databases and predicting their antibacterial activity via 1D NMR. Compared to traditional natural product discovery, this novel approach was time saving, accurate and promising [180]. Moreover, the omics approaches can also be used to predict the molecular target and biological mode of natural compounds, which was illustrated by the marine anticancer compound rhizochalinin [181] and marine sponge-associated bacteria [182].

## 4. Perspective of Marine-Derived Anticancer Compounds

Cancer remains an enormous threat to human health with its increasing morbidity and mortality [1]. Traditional cancer therapies, including surgery, radiotherapy and chemotherapy, together with advanced cancer therapies, such as immunotherapy, targeted therapy, gene therapy and vaccine therapy, have saved millions of lives. However, many people are still suffering because of tumor recurrence, drug resistance and treatment side effects. Hence, it is of great significance to discover novel effective and specific anticancer drugs.

Nature is a valuable source of multiple pharmaceuticals, and most of the anticancer drugs are natural products or derived from them. Oceans occupy about 70% of the earth’s surface and offer an exceptional environment for various marine organisms, bringing promising potential for novel drugs. Many marine-derived compounds were testified to contain multiple bioactivities, such as antibacterial, antifungal, antiviral, antituberculosis, antiprotozoal, antimalarial, anticoagulant, antioxidant and anticancer [6]. The discovery of novel anticancer drugs from marine samples is a popular, challenging but meaningful topic considering the current situation of cancer development. The first commercial anticancer marine drug was cytarabine, which has been applied in the clinical therapy of leukemia since 1969 [10]. After that, many compounds derived from marine organisms were introduced for cancer therapy. Among the recent anticancer drugs, ADCs emerged as a milestone for targeted therapy. Via the antibody of ADCs, it can selectively target the antigen-positive cancer cells and deliver the cytotoxic agent inside the malignant cells without hurting normal cells. Currently, most marine-derived anticancer drugs or potential anticancer drugs are ADCs, illustrating its significance in cancer therapy.

The advance and optimization of analyzing methods and technologies have greatly accelerated drug discovery, which is also applied to marine compounds. With increasing attention drawn to the ocean, together with the striking development of technology, there is no doubt that more and more candidates for cancer therapy will be discovered. Ultimately, we human beings will finally be the winners during this process.

## Figures and Tables

**Table 1:** Commercial marine-derived compounds.

Compound Name	Marine Organism	Chemical Class	Molecular Target (Target Hallmarks)	Cancer Type	References
Crytarabine	Sponge	Nucleoside	DNA polymerase	Leukemia	[8,9,10,11]
Eribulin mesylate	Sponge	Macrolide	Microtubules	Metastatic breast cancer	[8,9,12,13]
Brentuximab vedotin	Mollusk/ cyanobacterium	ADC (MMAE)	CD30 and microtubules	Anaplastic large T-cell systemic malignant lymphoma, Hodgkin disease	[8,14,15]
Trabectedin	Tunicate	Alkaloid	Minor groove of DNA	Soft tissue sarcoma and ovarian cancer	[8,16,17,18]
Plitidepsin	Tunicate	Dipsipetide	eEF1A2	Multiple myeloma, leukemia, lymphoma	[8,19,20,21,22]
Polatuzumab vedotin	Mollusk/cyanobacterium	ADC (MMAF)	CD76b and microtubules	Non-Hodgkin lymphoma, chronic lymphocytic leukemia, lymphoma, B-cell lymphoma, folicular	[8,23,24]
Enfortumab vedotin	Mollusk/cyanobacterium	ADC (MMAE)	Nectin-4	Metastatic urothelial cancer	[8,25]
Belantamab mafodotin	Mollusk/cyanobacterium	ADC (MMAF)	BCMA	Relapsed/refractory multiple myeloma	[8,26]
Lurbinectedin	Tunicate	Alkaloid	RNA polymerase II	Metastatic small-cell lung cancer	[8,27,28,29,30]

**Table 2:** Marine-derived compounds in Phase III status.

Compound Name	Marine Organism	Chemical Class	Molecular Target (Target Hallmarks)	Cancer Type	References
Marizomib	Bacterium	β-lactone-γ lactam	20S proteasome	Non-small-cell lung cancer, pancreatic cancer, melanoma, lymphoma, multiple myeloma	[8,37,38,39,40,41,42,43]
Plinabulin	Fungus	Diketopiperazine	Microtubules	Non-small-cell lung cancer, brain tumor	[8,44,45,46,47]

**Table 3:** Marine-derived compounds in phase II status.

Compound Name	Marine Organism	Chemical Class	Molecular Target (Target Hallmarks)	Cancer Type	References
W0101	Mollusk/ cyanobacterium	ADC (MMAE)	IGF-R1	Advanced or metastatic solid tumors	[8,49,50]
CX-2029	Mollusk/ cyanobacterium	ADC (MMAE)	CD71	Solid tumor, head and neck cancer, Non-small-cell lung cancer, pancreatic cancer, diffuse large B-cell lymphoma	[8,51,52,53]
CAB-ROR2	Mollusk/ cyanobacterium	ADC (MMAE)	ROR2	Solid tumor, non-small-cell lung cancer, triple-negative breast cancer, soft tissue sarcoma	[8,54]
RC48	Mollusk/ cyanobacterium	ADC (MMAE)	HER2	Urothelial carcinoma, advanced cancer, gastric cancer, HER2-overexpressing gastric carcinoma, advanced breast cancer, solid tumors	[8,55,56,57]
Enapotamab vedotin	Mollusk/ cyanobacterium	ADC (MMAE)	Axl RTK	Ovarian cancer, cervical cancer, endometrial cancer	[8,58,59,60]
Telisotuzumab vedotin	Mollusk/ cyanobacterium	ADC (MMAE)	c-Met	Solid tumors	[8,61,62,63,64]
Ladiratuzumab vedotin	Mollusk/ cyanobacterium	ADC (MMAE)	LIV-1 and microtubules	Breast cancer	[8,65,66,67]
Tisotumab vedotin	Mollusk/ cyanobacterium	ADC (MMAE)	Tissue factor and microtubules	Ovary cancer, cervix cancer, endometrium cancer, bladder cancer, prostate cancer (CRPC), cancer of head and neck (SCCHN), esophagus cancer, lung cancer (NSCLC)	[8,68,69]
AGS-16C3F	Mollusk/ cyanobacterium	ADC (MMAF)	ENPP3 and microtubules	Renal cell carcinoma	[8,70,71,72]
Plocabulin	Sponge	Polyketide	Minor groove of DNA	Solid tumors	[8,73,74,75,76]

**Table 4:** Marine-derived compounds in phase I status.

Compound Name	Marine Organism	Chemical Class	Molecular Target (Target Hallmarks)	Cancer Type	References
MORAb-202	Sponge	ADC (macrolide)	Microtubules	Solid tumors	[8,88,89]
XMT-1536	Mollusk/ cyanobacterium	ADC (dolaflexin)	NaPi2b and microtubules	Solid tumors	[8,90,91,92,93,94]
RF06804103	Mollusk/ cyanobacterium	ADC (auristatin variant)	HER2	Breast neoplasms, stomach neoplasms, esophagogastric junction neoplasm, carcinoma, non-small-cell lung cancer	[8,95,96,97,98]
ARX-788	Mollusk/ cyanobacterium	ADC (MMAE)	HER2 and microtubules	Breast cancer, gastric cancer	[8,99,100,101]
ALT-P7	Mollusk/ cyanobacterium	ADC (MMAE)	HER2 and microtubules	Breast cancer, gastric cancer	[8,102,103]
ZW49	Mollusk/ cyanobacterium	ADC (auristatin variant)	HER2	HER2-expressing cancers	[8,104]

**Table 5:** Technologies in marine bioactive compound discovery.

Technology	Procedure	Principle	Development/Application in Marine Compounds	References
Supercritical fluid extraction (SFE)	Extraction/ separation	The supercritical fluids generated by CO_2_ increase the sample’s dissolution via its potent diffusion inside the sample	Combined with pre-treatment to extract lipids from marine diatom	[137,138,139,140]
Pressurized liquid extraction (PLE)	Extraction/ separation	High pressure (50–300 psi) and high temperature (50–200 °C) enable effective penetration and solubility of the solutes	Conditional PLE to obtain antioxidant protein from sea bass	[141,142,143]
Enzyme-assisted extraction (EAE)	Extraction/ separation	Increase extraction yield without changing their features via its biocatalysts	Combined with other extracting methods to extract proteins from seaweed	[144,145,146,147,148]
Ultrasound-assisted extraction (UAE)	Extraction/ separation	Cavitation of ultrasonic waves provides a stronger penetration of solvent and straightforward disruption of cell membranes	Combined with maceration or homogenization to extract phycobiliproteins from macroalgae	[149,150]
Microwave-assisted extraction (MAE)	Extraction/ separation	Via microwave absorption, heat is generated within the whole material, causing dilapidation	MAE–DLLME -GC/MS to extract and analyze PAHs in smoked fish	[151,152,153,154]
Solid phase microextraction (SPME)	Extraction/ separation	Based on the partition equilibrium of the extractives’ stationary phase generated by a fiber connected with extracting phase	SPME-GC/MS to extract biogenic amines from fish	[155,156,157]
Solid phase extraction (SPE)	Extraction/ separation	Using a solid phase to absorb the desired compounds from the sample	Anionic exchange SPE to extract the organic acids from microbial samples; SPE-NMR to analyze oil-in-water content in water	[158,159]
Vibrational spectroscopy (VS)	Structure characterization	Measure the spectroscopy of vibration generated by absorption or emission of electromagnetic radiation	scRS-1DCNN to identify individual marine microorganisms	[160,161,162,163,164]
Nuclear magnetic eesonance spectroscopy (NMR)	Structure characterization	Analysis of the spectroscopy generated by specific magnetic properties around different atomic nuclei	PULCON-qNMR to quantify marine toxins; benchtop NMR to characterize enzymatic hydrolysis reactions in red cod, salmon and shrimp	[165,166,167,168]
Mass spectrometry (MS)	Structure characterization	Define the elemental or isotopic signature of a sample via its mass spectrum (mass-to-charge ratio of ions)	MALDI-TOF MS to identify protein protease from marine invertebrate extracts (fast and sensitive); GC–QQQ-MS to identify Steryl glycosides in marine microalgae; HPLC-MS to elucidate palytoxin congener from the marine dinoflagellate; LLE-TLC-MS/MS to separate and analyze marine toxins. ESI-HILIC-MS to identify As-PL in marine algae; UHPLC-MS to screen the enzyme inhibitors on marine natural products	[169,170,171,172,173,174,175]
High-throughput antimicrobial screening	Bioactivity screening	Analyze antimicrobial ability via an automatic yeast model system	Automatic and high-throughput antimicrobial screening for natural products (including marine sponge extracts and fungal extracts)	[176]
Antibiotic mode of action profile (BioMAP) screening	Bioactivity screening	Automatically pin the compounds into a 384-well plate, which contains pathogenic strains, and read the absorbance automatically every hour	Automatic, accurate and efficient antibiotic screening for natural products (including marine products)	[177]
Omics screening	Bioactivity screening	Synergetic analysis of genomics, transcriptomics, proteomics and metabolomics	Genomic for antinematode compounds from marine bacterium; proteomic for action mode of marine anticancer compound rhizochalinin	[178,179,180,181,182]
